# The Human Brain Encodes a Chronicle of Visual Events at Each Instant of Time Through the Multiplexing of Traveling Waves

**DOI:** 10.1523/JNEUROSCI.2098-20.2021

**Published:** 2021-08-25

**Authors:** Jean-Rémi King, Valentin Wyart

**Affiliations:** ^1^Frankfurt Institute for Advanced Studies, 60438, Frankfurt, Germany; ^2^New York University, New York, New York, 10012; ^3^Laboratoire des Systèmes Perceptifs (Centre National de la Recherche Scientifique Unité Mixte de Recherche 8248), Département d'Études Cognitives, École Normale Supérieure, Université Paris Sciences et Lettres, 75005, Paris, France; ^4^Laboratoire des Neurosciences Cognitives et Computationnelles (Institut National de la Santé et de la Recherche Médicale U960), Département d'Études Cognitives, École Normale Supérieure, Université Paris Sciences et Lettres, 75005, Paris, France

**Keywords:** decoding, dynamical system, EEG, streams, time, visual perception

## Abstract

The human brain continuously processes streams of visual input. Yet, a single image typically triggers neural responses that extend beyond 1s. To understand how the brain encodes and maintains successive images, we analyzed with electroencephalography the brain activity of human subjects while they watched ∼5000 visual stimuli presented in fast sequences. First, we confirm that each stimulus can be decoded from brain activity for ∼1s, and we demonstrate that the brain simultaneously represents multiple images at each time instant. Second, we source localize the corresponding brain responses in the expected visual hierarchy and show that distinct brain regions represent, at each time instant, different snapshots of past stimulations. Third, we propose a simple framework to further characterize the dynamical system of these traveling waves. Our results show that a chain of neural circuits, which each consist of (1) a hidden maintenance mechanism and (2) an observable update mechanism, accounts for the dynamics of macroscopic brain representations elicited by visual sequences. Together, these results detail a simple architecture explaining how successive visual events and their respective timings can be simultaneously represented in the brain.

**SIGNIFICANCE STATEMENT** Our retinas are continuously bombarded with a rich flux of visual input. Yet, how our brain continuously processes such visual streams is a major challenge to neuroscience. Here, we developed techniques to decode and track, from human brain activity, multiple images flashed in rapid succession. Our results show that the brain simultaneously represents multiple successive images at each time instant by multiplexing them along a neural cascade. Dynamical modeling shows that these results can be explained by a hierarchy of neural assemblies that continuously propagate multiple visual contents. Overall, this study sheds new light on the biological basis of our visual experience.

## Introduction

The human visual system is continuously bombarded with a flux of visual input. To interact with its environment, our brain must, continuously transform these visual events into abstract representations (Tenenbaum et al., 2011), track their relative motions (Goldstein and Brockmole, 2016; Born and Bradley, 2005), and resolve countless ambiguities (Knill and Pouget, 2004). Yet, electrophysiology and neuroimaging studies have primarily focused on the brain responses to static images (although see Nishimoto et al., 2011; VanRullen and Macdonald, 2012; Marti and Dehaene, 2017; van Vugt et al., 2018). The resulting studies consistently show that flashing an image onto the retina leads to cortical responses that often last up to 1s (Carlson et al., 2013; Cichy et al., 2014; King et al., 2016). The timing and location of these neural activations are consistent with a hierarchical inference network (Friston, 2010; Cichy et al., 2014; Yamins et al., 2014; Poggio and Anselmi, 2016; Gwilliams and King, 2020) and suggest that the primary visual cortex encodes low-level representations (e.g., luminosity and orientations of visual edges), whereas the inferotemporal and dorsoparietal cortices encode abstract representations (e.g., the presence of a face in the visual field).

The neural dynamics revealed in these studies thus highlight a fundamental paradox: the duration of visual processing (∼1000 ms) can largely exceed the duration of sensory stimulation (e.g., 17 ms in King et al., 2016). Consequently, it is unclear how the brain copes with *streams* of sensory inputs: that is, how can we simultaneously maintain successive visual representations without mixing their ordering?

**Figure 1.**
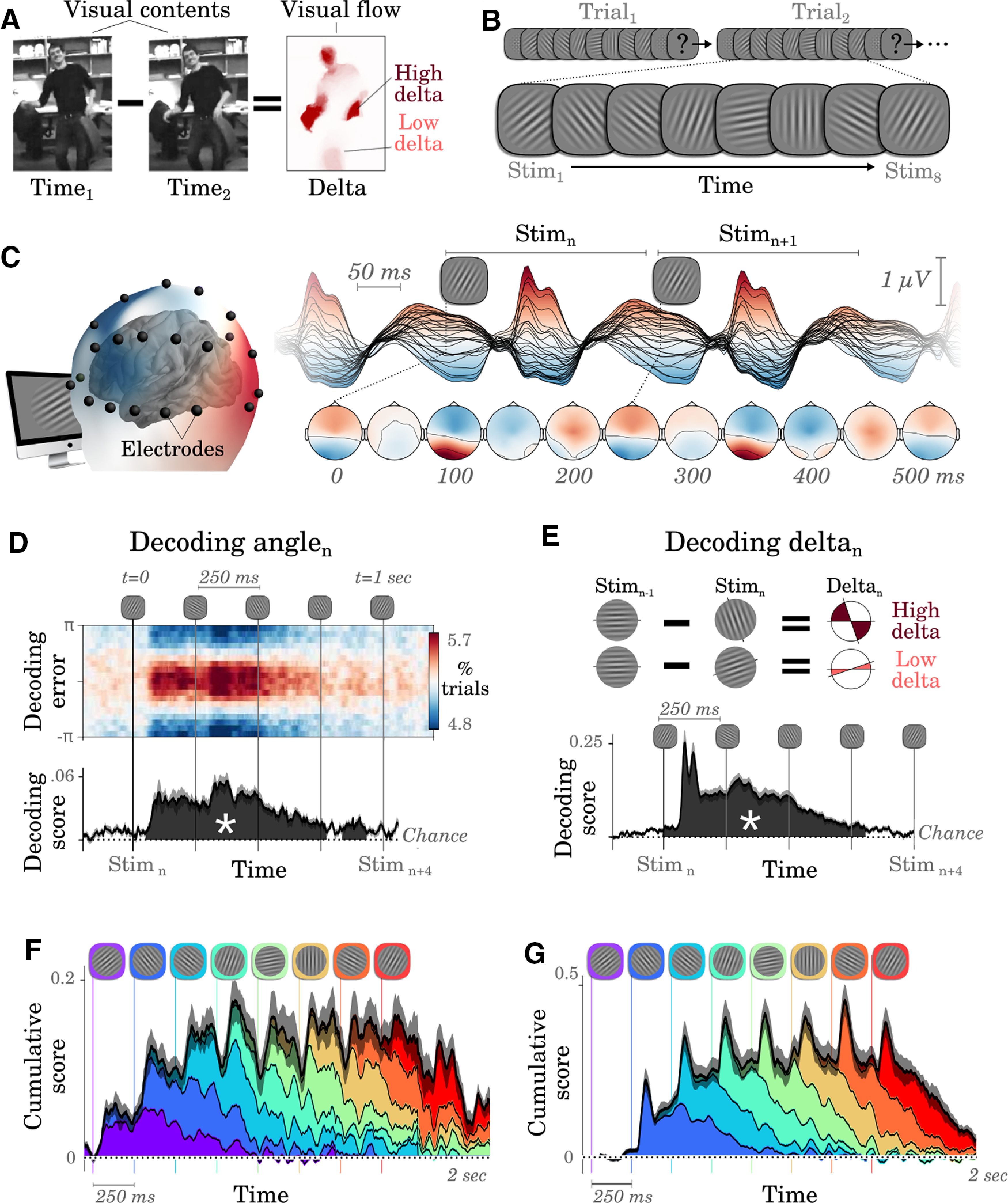
Successive images are simultaneously represented in brain activity. ***A***, Visual contents refer to what is in the image at time (*t*). Visual flow refers to the amount of change between *t*_1_ and *t*_2_. ***B***, Subjects watched ∼5000 randomly oriented Gabor patches, flashed every 250 ms and grouped into 8-item sequences separated by masks. Each sequence ended in a two-alternative forced choice where subjects indicated whether stimuli fell, on average, closer to the cardinal or diagonal axes. ***C***, Brain activity was recorded with EEG. Each line shows the average response evoked by the stimuli. ***D***, Top, Distribution of single-trial decoding error of Stim_n_ as a function of time relative to the onset of stimulus *n*. Bottom, Time course of the corresponding decoding score. The shaded regions (with an asterisk) indicate significant decoding across subjects (cluster corrected). ***E***, Decoding scores of visual flows (approximated as the absolute angular difference between successive stimuli) as a function of time relative to stimulus onset. ***F***, Cumulative decoding scores (black) and the contribution of each of the eight successive stimuli (color-coded by position in the 8-item sequence), as a function of time relative to the sequence onset (chance = 0). ***G***, Similar to ***F*** for cumulative δ decoding scores. In panels ***C***–***G***, the vertical lines mark the onsets of each stimulus. Error bars indicate the SEM across subjects.

To address this question, we recorded the human brain activity with source-localized electroencephalography (EEG) while subjects watched ∼5000 parametrically controlled Gabor patches flashed in rapid succession and grouped into 8-stimulus trials (Fig. 1*B*,*C*). Unlike natural movies, this paradigm allows us to fully decorrelate successive images, which, in turn, allows us to identify which visual event each brain region encodes at a given time instant. Our study provides three main contributions. First, we confirm with whole-brain decoding analyses that the human brain simultaneously represents multiple images that have been presented sequentially. Second, we show with temporal generalization and source analyses that each stimulus triggers a cascade of representations that propagates across the expected visual hierarchy. Finally, we implement a principled search of dynamical systems and identify the simplest neural architectures that can account for these EEG findings. Overall, our results show that a simple neural hierarchy can explain how the brain dynamically processes and maintains successive visual inputs without confusing their ordering.

## Materials and Methods

### 

#### 

##### Subjects

Sixteen healthy adults (age, 18–25 years of either sex), all with normal or corrected-to-normal vision and no reported history of neurologic or psychiatric disorders, were recruited from the University of Oxford. Subjects provided written consent before participating in the experiment and received £30 in compensation for their participation. Approximately £5 of bonuses could be additionally obtained depending on subjects' performance on the two-alternative forced-choice task. The study followed local ethics guidelines. The data from one participant was excluded because of eye artifacts. The investigation of subjects' decisions at the end of each sequence is reported in Wyart et al. (2012).

##### Procedure

The stimuli were displayed on a CRT monitor (1024 × 768 pixels, refreshed at 60 Hz) placed ∼80 cm away from subjects' eyes, and controlled with the MATLAB (MathWorks) Psychophysics Toolbox version 3 (Brainard, 1997; Pelli, 1997). Each trial consisted of a sequence of 11 successive visual stimuli flashed every 250 ms. The first two items and the last item were task-irrelevant masks, generated from the average of four cardinal and diagonal Gabor patterns and were not considered in the present analyses. The purpose of these masks was to increase the homogeneity across the stimuli; for example, the first Gabor patch would already be presented in a stream. The remaining eight stimuli (hereafter referred to as the 8-item sequences) were Gabor patches with fixed contrast (50%), diameter (4° of visual angle), spatial frequency (2 cycles per degree of visual angle), and envelope (Gaussian with a SD of 1° of visual angle). Stimulus orientations, however, varied following a uniform distribution across all trials. One blank frame (16.7 ms) was introduced before the onset of each stimulus to avoid visual tearing artifacts. The intertrial interval was 1250 s (±250 ms). The experiment consisted of 672 trials, divided into 7 sessions of 96 trials, and thus consisted of a maximum of 5376 usable brain responses to oriented stimuli over approximately one hour of recording.

##### Task

To ensure subjects paid attention to the visual stimuli, they were asked to report after each 8-stimulus sequence whether the orientation of the stimuli was, on average, closer to (1) the cardinal axes (horizontal or vertical) to (2) the diagonal axes (45° or 135°). Responses were given by pressing their left or right index finger on either the right or left CTRL buttons (e.g., diagonal = left finger, cardinal = right finger), with a response mapping counterbalanced across subjects. This two-alternative forced-choice task was adapted for each subject to homogenize attention and performance across subjects. Specifically, the orientation of each Gabor patch was distributed uniformly across all trials but drawn, within each sequence, from a probability density function adapted for each subject's performance. To this end, subjects performed a practice and a titration session before the main experiment to estimate their 75% accuracy psychophysical threshold with an adaptive staircase procedure (Kaernbach, 1991). This psychophysical threshold served to determine three evenly-spaced difficulty levels. For example, an easy sequence would consist of stimuli whose orientations tend to fall close to the cardinal axes, whereas a hard sequence would consist of stimuli whose orientations tended to fall in between the cardinal and diagonal axes. Easy and difficult trials (one-third of all trials each) had a categorization sensitivity of 2.12 (SEM ±0.18) and 1.00 (SEM ±0.09), respectively. Neutral trials (one-third of all trials) were associated with a pseudorandom feedback, positive on 60% of neutral trials. Additional behavioral and brain correlates of subjects' decision are reported in Wyart et al. (2012).

##### EEG acquisition and preprocessing

EEG signals were recorded with a 32-channel Neuroscan system and a Synamps 2 digital amplifier (Neuroscan Compumedics). In addition, the horizontal and vertical electrooculograms were recorded with four bipolar-mounted electrodes. Electrode impedances were kept below 50 kΩ. EEG signals were recorded at 1000 Hz and high-pass filtered online at 0.1 Hz and later low-pass filtered at 40 Hz, down-sampled to 250 Hz, segmented from 500 ms before the onset of the first stimulus (the premask) to 1s following the offset of last stimulus (the postmask). These epochs were visually inspected (1) to remove trials containing nonstereotypical artifacts and (2) to identify artifacted electrodes. In total, three participants had a single bad electrode, which was consequently interpolated to the weighted average of neighboring electrodes.

##### Analyses

The stimulus orientation is here used to test whether brain activity represents (i.e., linearly correlates with) the visual content present on the retina at a given instant. Because stimulus orientation is circular, the encoding and decoding analyses of stimulus angles are based on the linear regression of (1) the EEG and (2) the sine and cosine of the stimulus angles (horizontal = 0 radian; vertical = 2π radian). By contrast, the change of orientation (δ = |angle*_n_* − angle*_n_*_-1_|) is here used as a way to probe whether brain activity codes for visual flow (Fig. 1*A*). The first Gabor patch of the sequence was ignored from δ analyses.

##### Decoding

Multivariate linear decoding models were implemented following a three-step procedure: fitting, predicting, scoring within subjects. Specifically, for each subject separately we fitted an ordinary least square regression across all EEG channels recorded at a given time sample *t* relative to the onset of stimuli *n* to predict the features of stimulus *n* (i.e., angle and δ) following the methods of (King et al., 2016). Each decoder thus consisted of a linear spatial filter ($W_{t}\in {ℜ}^{m\times 3}$) optimized as follows:

$$W_{t}={\left(X_{t}\prime X_{t}\right)}^{-1}X_{t}\prime Y_{n},$$

where $X_{t}\in {ℜ}^{q\times m}$ corresponds to the *q* trials of *m* electrodes recorded at time *t* after the onset of the stimulus, $Y_{n}\in {ℜ}^{q\times 3}$ corresponds to the δ between stimuli *n* and stimuli *n-1*, the sine and the cosine of stimuli *n*, and ′ represents the transpose operator. Each spatial filter *W_t_* was then used to predict the angles and the deltas of out-of-sample EEG data (see cross-validation) as follows:

$${\hat{Y}}_{t}=X_{t}\cdot W_{t},$$

where ${\hat{Y}}_{t}$ represents the estimated angle (sin and cosine) and the estimated δ of each out-of-sample EEG recording at time *t*.

Finally, δ decoding scores were summarized with a Pearson's correlation coefficient *r* between the out-of-sample δ predictions and the corresponding true δ. Angle decoding scores were summarized by computing the angular difference between the true angles (α) and the out-of-sample angular predictions ($\hat{\alpha }=arctan2\left({\hat{Y}}_{sin},{\hat{Y}}_{cos}\right)$). For clarity, this angular error is reported throughout the manuscript as an angular score ($score=\pi /2-\alpha -\hat{\alpha }$; chance = 0). The decoders were trained within each subject with a cross-validation scheme across all stimuli independently of their position in a given sequence.

To interpret the decoders, we transformed the spatial filters *W* into spatial patterns *P* following Haufe's method (Haufe et al., 2014) estimated as follows:

$$P=\Sigma_{X}W\Sigma_{\hat{Y}}^{-1},$$

where Σ*_X_* and Σ*_Ŷ_* refer to the empirical covariances of *X* and *Ŷ* respectively. Because we use linear regression, the expected value of Haufe's patterns will be the following encoding coefficients: $\Sigma_{X}W\Sigma_{Y}^{-1}=\Sigma_{X}\Sigma_{X}^{-1}X\prime Y\Sigma_{Y}^{-1}=X\prime Y{\left(Y\prime Y\right)}^{-1}$. Consequently, the present decoders are not black-boxes but directly relates to a standard encoding analysis, while maximizing signal-to-noise ratio.

##### Temporal generalization

Temporal generalization (TG) analysis consists in testing the ability of each temporal decoder to generalize over all time samples (Stokes et al., 2013; King and Dehaene, 2014). Specifically, each decoder trained at a given time *t* is scored on its ability to decode brain activity from a time *t′*. A significant temporal generalization suggests that the brain activity patterns observed at time *t* are partially similar to those observed at time *t′*. Because our decoders are linear, and because the EEG activity reflects a linear projection of the neuronal activity onto our sensors, a significant generalization suggests that a similar population of neurons is activated at time *t* and *t′*. Conversely, if two decoders trained at *t* and *t′*, respectively, can both be used to decode the EEG activity at the respective training time but fail to cross-generalize with one another, then this suggests that the populations of neurons activated at *t* and *t′* significantly differ. Consequently, TG results in a training by testing time matrix, which can be used as a method to characterize the spatiotemporal dynamics of the coding response (Stokes et al., 2013; King and Dehaene, 2014).

##### Encoding

To assess the extent to which visual content and visual flow comparatively predict subjects' EEG, we applied encoding analyses for each subject separately. Specifically, we fit, for each electrode and each time-sample separately, an ordinary least-square regression to predict the amplitude of each electrode at each time sample from our experimental variable *Y* as follows:

$$P_{c,t}={\left(Y_{n}\prime Y_{n}\right)}^{-1}Y_{n}\prime X_{c,t}$$

$$\hat{X}=YP$$

We summarize the encoding results with Pearson's correlation coefficient *r* obtained for each electrode and each time sample relative to the stimulus onset, between the true voltage and the voltage predicted by our model. Using cross-validation, we assessed whether these predictions correlated with the actual EEG voltage.

##### Cross-validation

Decoding and encoding analyses were implemented within an *ad hoc* stratified *K*-fold cross-validation procedure split across 8-item sequences. Specifically, cross-validation was designed to ensure that two stimuli from the same 8-item sequences never appeared both in the training and testing sets.

##### Source estimates

**Figure 2.**
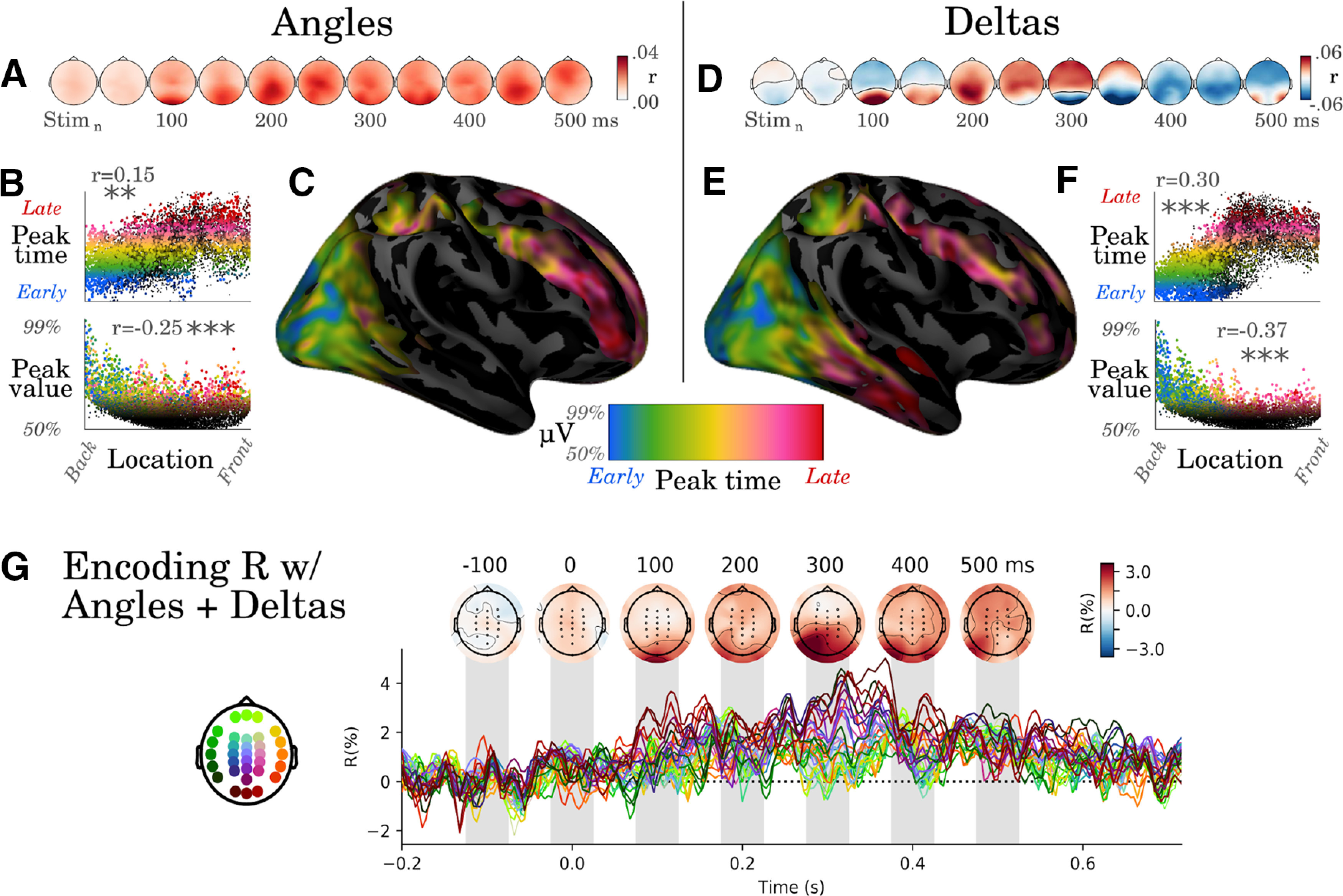
Visual representations propagate from sensory to associative cortices. ***A***, Correlation scores resulting from encoding analyses, trained to predict the EEG activity from the sine and cosine of the stimulus angles. ***B***, Each dot corresponds to a source estimated from the EEG coding topographies with a minimum norm estimation. The *x*-axis corresponds to the source location along the posteroanterior direction. Top, The *y*-axis either indicates the relative timing of the peak activity in each source or Bottom, The intensity of this peak. Asterisks indicate statistical significance (**p < 0.01, ***p < 0.001) ***C***, Same data as ***B*** but plotted on the cortical surface. Colors indicate both the peak amplitude (e.g., black: amplitude = median amplitude across sources) and the peak latency (e.g., blue: peak within analogous to the 5% percentile of the earliest responses across sources; red: peak beyond 95% percentile). ***D***, Correlation coefficients between deltas and EEG amplitude. ***E***, ***F***, Analogous analyses tp B-C applied to the brain responses coding for the changes between successive stimuli (δ). ***G***, Cross-validated encoding scores (Pearson's *r*) obtained with both angles (sin + cos) and deltas. Colors indicate EEG channels. The results can be visualized interactively at https://kingjr.github.io/chronicles/.

The locations of the neural sources corresponding to effects observed at sensor levels were estimated following MNE source reconstruction pipeline (Gramfort et al., 2014). The noise covariance was estimated from the 200 ms baseline activity preceding each 8-item sequence. The forward model derived from FreeSurfer's fsaverage 3-layer mesh and manually coregistered with the 32 scalp electrodes. The inverse model was fitted with a minimum norm estimate with MNE default parameters (λ^2^ = 0.125, free dipole with normal component). The peak amplitudes and latencies (Fig. 2*B*,*C*,*E*,*F*) were computed from the relative amplitude and relative latency of the maximal amplitude obtained for each source and each subject separately. The corresponding figures show these effects averaged across subjects.

##### Statistics

Except if stated otherwise, all inferential statistical estimates derive from two-tailed second-level nonparametric analyses across subjects. Specifically, each decoding, encoding, and source analysis was applied within each subject separately and led to a unique estimate of the effect size obtained across time samples (e.g., an *r* correlation coefficient for each subject). A second-level analysis was then applied across subjects to assess whether the distribution of these effect sizes was different from chance. This second-level analysis was either (1) a Wilcoxon test applied across subjects (in the case of a nonrepeated analysis) or (2) a spatiotemporal cluster test applied across subjects (in the case of repeated measurements, such as decoding time courses or encoding spatiotemporal effects). The *p* values of the decoding time-courses, TG matrix, sources estimates, and EEG topographies all refer to the *p* value of the significant spatiotemporal cluster (Maris and Oostenveld, 2007) as implemented in MNE with default parameters (Gramfort et al., 2014). The error bars plotted in the figures correspond to the SEM across subjects.

##### Modeling neural dynamics

We used a discrete-time dynamical system framework to simulate the spatiotemporal dynamics evoked by one or several successive stimulations of fixed duration. Each model consists of a set of units *z*, which each transforms the sum of their inputs $u\in {ℜ}^{t}$ with a potentially nonlinear activation function (*f*):

$$z\left(t+1\right)=f_{z}\left(\sum uw(z,u)\cdot u(t)\right),$$

where *t* refers to the time sample and *w*(*z, u*) refers to the connection from unit *u* to unit *z*. Except if stated otherwise, *f* is the identity activation function (i.e., a linear function: *f*(*z*) *= z*). Each architecture consisted of a repeated pattern of connections between layers, each consisting of observable (x) and/or hidden (*y*) units connected through recurrent [*w*(*x_i_*, *x_i_*), *w*(*y_i_, y_i_*), *w*(*x_i_*, *y_i_*), *w*(*y_i_, x_i_*)], feedforward [*w*(*x_i_*, *x_i_*_+*1*_), *w*(*y_i_, y_i+1_*), *w*(*x_i_, y*_*i*+*1*_), *w*(*y_i_, x_i_*_+*1*_)], and feedback connections [*w*(*x_i_*, *x_i_*_−*1*_), *w*(*y_i_, y_i-1_*), *w*(*x_i_, y_i-1_*), *w*(*y_i_, x_i_*_−*1*_), see Fig. 4*C*]. In other words, each unit can only be connected to other units within the same layer (1), the layer above (i + 1) or the layer below (i − 1) as follows:

$$z\left(t+1\right)=f_{z}\left(\sum_{j=i-1}^{i+1}(w\left(z,xj\right)\cdot x_{j}\left(t\right)+w\left(z,yj\right)\cdot y_{j}\left(t\right)\right)).$$

The simulated activity was analyzed with decoding and temporal generalization analyses after a linear random projection ($F\in {ℜ}^{d_{x}\times 32}$, normally distributed around 0, with SD = 1) of the *x* units onto 32 noisy virtual sensors *X* as follows:

$$X\left(t\right)=Fz_{x}\left(t\right)+\varepsilon$$,

where *X*(*t*) is the activity of EEG channels at time *t*, *z_x_* is the activity of observable units, and ε is a Gaussian noise (except if stated otherwise, SD = 1).

This random projection and the subsequent decoding and TG analyses were repeated 15 times to mimic the analyses of 15 subjects.

Except in Figure 2*A*, units do not intend to simulate individual neurons but aim to approximate the dynamical characteristics of the macroscopic electric field. In this view, some recurrent connections may be equally interpreted as an adaptation mechanism (i.e., activity reduction caused by a cellular mechanism) or as a lateral inhibition mechanism (i.e., activity reduction caused by an intercellular mechanism). In either case, the hidden units *y* are designed to account for the possibility that some neural dynamics may be influenced by mechanisms that cannot be directly observed with EEG (e.g., adaptation does not generate an electric field, and the inconsistent orientations of the interneurons' electric fields are not easily detected from distant EEG electrodes).

##### Architecture search

To identify the architectures that could account for our empirical observations, we (1) implemented a grid-search analysis over architectures, connection weights, and activation functions and (2) tested whether the resulting dynamics consisted of a similar spatiotemporal response.

1. The search considered hierarchical networks with one observable unit (*x*) and one hidden unit (*y*) per level *i*. Consequently, there are four possible recurrent recurrent, four possible feedforward, and four possible feedback possible connections (see Fig. 4*C*). An architecture is the set of models whose connections have identical signs. For example, two models A and B, which each consists of a unique feedforward connection between unit *x_i_* and unit *x*_*i*+*1*_ and whose weight is 0.75 and 1.00, respectively, belong to the same architecture; that is, a positive feedforward architecture. For each connection, we tested 21 possible values, linearly distributed between −1 and 1. Finally, to extend our model search to nonlinear dynamics, we also search across four monotonic activation functions: Linear: *f*(*z*) *= z;* Relu: *f*(*z*) *=* max(*z*, *0*)*;* SatRelu: *f*(*z*) *=* min(max(*z*, *0*), *1*) and SatLin: *f*(*z*) *=* min(max(*z*, −*1*), *1*). The above activation functions were applied independently for *x* and *y* units. Overall, the total search could thus span up to 4^2^ activation functions by 21^12^ connection weights, that is, more than 10^17^ distinct models. To find the simplest models that account for our EEG results, we searched for valid models with an increasing complexity (i.e., first with only one connection, then two, then three). We stopped when at least one model was found at a given complexity level, while making sure all models at that level of complexity were evaluated. Given that the search found valid models with four connections, we simulated, in total, ∼1.5B models. All of the tested models were implemented with 10 hierarchical levels and simulated over 120 time samples, with a constant input between time 30 and 60. The input was connected to the network following the feedforward weights.

2. Each model was assessed on its ability to account for the three main findings identified in our EEG study: 2.1 Stimulus onset evokes a transient traveling wave (onset), 2.2 Stimulus offset evokes a transient traveling wave with opposite amplitude (offset), and 2.3 The phase (i.e., width) of these waves increases across levels (increasing maintenance). We quantified these properties for the observable units *x*. With the *y* units being hidden, they could thus have any dynamical response.

2.1. Onset. A unit was considered to be marked by an onset if its maximum value *M* reached at time *t_M_* was positive, if *t_M_* was before stimulus offset, and if all values between stimulus onset and *t_M_* increased over time. A network was considered to generate an onset traveling wave if all the x units were marked by an onset at each level.

2.2. Offset. A unit was considered to be marked by an offset if its minimum value *m* reached at time *t_m_* was negative and if all values between *m* and the end of the simulation increased toward 0. A network was considered to generate an offset traveling wave if all the *x* units were marked by an offset at each level.

2.3. Increasing maintenance. A maintenance half-life was estimated for each level by estimating by the delay *t_h_* it takes a unit to reach half of its maximum value *M*: $x\left(t_{h}\right)=x\left(t_{M}\right)/2$. Half-lives were estimated if all values between *t_M_* and *t_m_* decreased toward 0. The network was considered to have increasing maintenance if half-lives increased across levels as follows: $\frac{dh}{di}\geq 0$.

## Results

### Successive visual stimuli can be simultaneously decoded at each time instant

To track the brain representation of each individual stimulus within a visual sequence, we decoded (1) the orientation of each Gabor patch and (2) the angular difference between two successive Gabor patches, that is, a dominant and orthogonal signature of (1) optical content and (2) optical flow, respectively (Stocker and Simoncelli, 2006; Wang et al., 2016; Fig. 1*A*).

EEG signals linearly correlated with both the orientation of each stimulus *n* (angle*_n_*) and the absolute change between successive stimuli (delta*_n_* = |angles*_n_*_+1_–angle*_n_*|; deltas and angles are orthogonal by design). Specifically, we fit multivariate linear regressions (*W_t_*) at each time sample *t* relative to the onset of each stimulus to predict the sine and cosine of its angle given the voltage of all EEG electrodes. We then assessed with cross-validation whether these predicted angles correlated with the true stimulus angles. This angle decoding was significantly above chance across subjects between ∼50 and ∼950 ms after stimulus onset (cluster-corrected effects across subjects illustrated in Fig. 1*D*).

We applied an analogous analysis to decode the change of orientations between successive pairs of stimuli (δ) to assess whether and when brain activity represented optical flows. The corresponding δ decoding time course was similar to the angle decoding time course (Fig. 1*E*).

Decoding analyses, assessed for each successive stimulus within a visual stream, revealed that multiple stimuli are simultaneously represented at each time sample (Fig. 1*F*,*G*). On average, between two and five angles and between two and four deltas can be simultaneously decoded at each time point, although decoding precision rapidly diminishes with time (linear regression between scores and stimulus distance: β_Angles_ = 0.007, *p* = 0.001; β_δ_ = 0.028, *p* < 0.001).

### The propagation of representations is localized along the ventral and dorsal visual pathways

Do these sustained decoding performances relate to a sustained activity pattern in the brain? To test this issue, we analyzed the spatial patterns associated with angle and δ representations using encoding analyses. For both angle and δ analyses, the spatial patterns peaked around occipital electrodes and subsequently propagated to anterior electrodes (Fig. 2*A*,*D*). The source estimates of these EEG topographies suggest that the earliest neural responses coding for both angles and deltas are generated in the early visual cortex and are subsequently followed by responses in the inferotemporal and dorsoparietal cortices (Fig. 2,*B*,*C*,*E*,*F*). Furthermore, neural responses coding for deltas reached significance in the superior frontal cortex ∼600 ms after stimulus onset. Finally, a full decomposition of the brain responses into their relative peak latencies and peak amplitudes confirmed the overall occipitofrontal propagation of the representations of stimulus angles (*r* = 0.15, *p* < 0.01) and stmulus deltas (*r* = 0.30, *p* < 0.001, Fig. 2*B*,*C*,*E*,*F*). Overall, although EEG source reconstruction should be interpreted with caution, our estimates confirm that visual stimuli trigger a traveling wave across the visual pathway (Riesenhuber and Poggio, 1999; van Vugt et al., 2018) and further suggest that this traveling wave encodes low-level visual information (i.e., the orientation of Gabor patches and their change).

### A simple framework to formally characterize neural architectures and their dynamics

**Figure 3.**
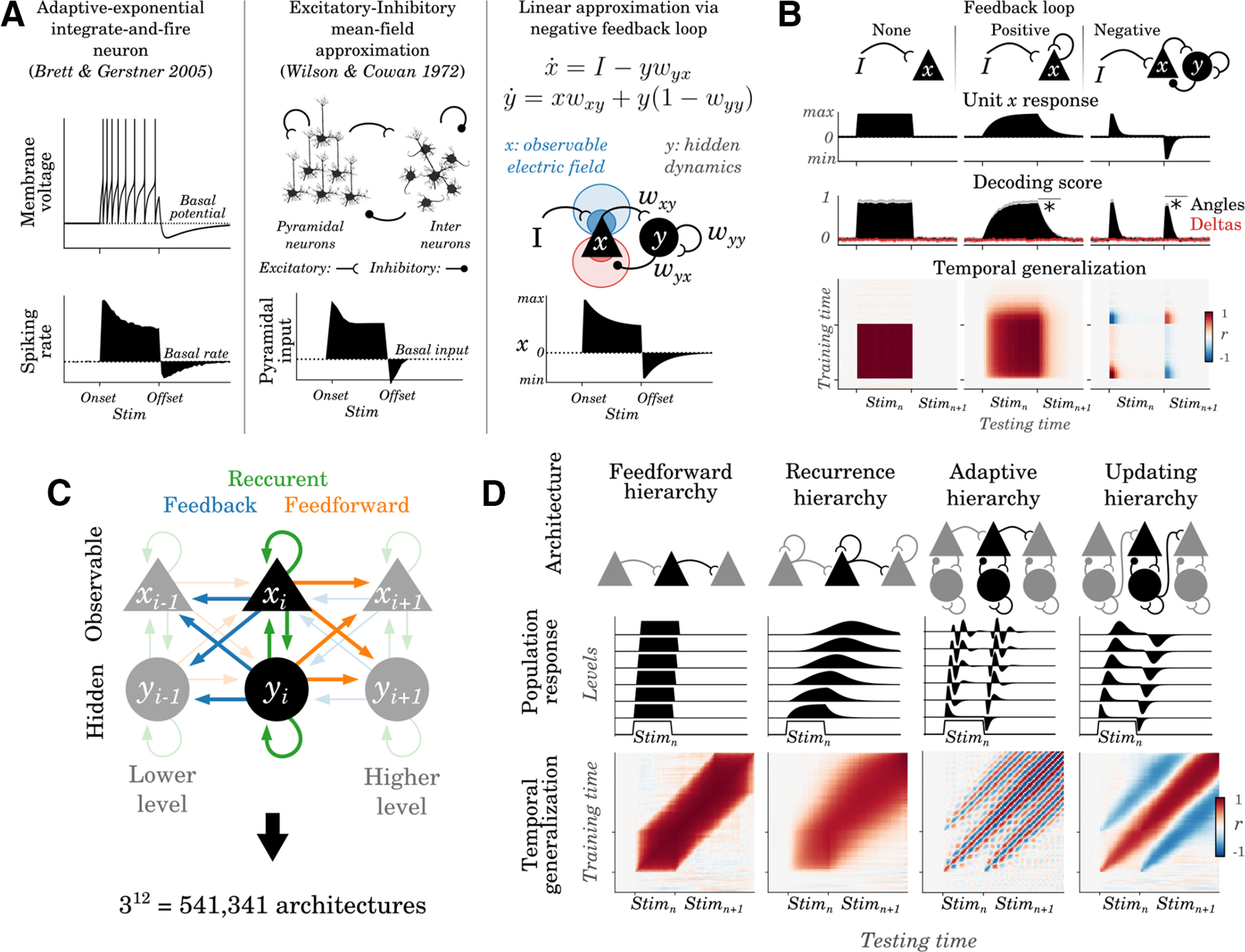
Dynamical system framework. ***A***, Left, The membrane potential (top) and the expected spike rate (bottom) of an AdexpIAF neuron in response to a sustained input (onset and offset indicated by the ticks); the dotted lines indicate the basal activity at rest. Middle, In an excitatory/inhibitory population of neurons stimulated with an input, the alignment of pyramidal dendrites leads their PSP to be detectable from distant electrodes, whereas the interneurons of PSP are not detectable with EEG. Right, A linear dynamical system composed of two units (*x* = observable; *y* = hidden) connected in a feedback loop can approximate adaptive neurons or Excitatory/Inhibitory (E/I) balance responses: *x* captures an observable variable (e.g., the electric field associated with spiking activity or pyramidal PSP), whereas *y* captures a hidden variable (e.g., ion currents associated with adaptation or inhibitory PSP). ***B***, Columns illustrate the predictions of no, positive and negative, and feedback loop circuits, respectively. The top black line illustrates the activity of an observable unit (*x*) in response to a stimulus (onset and offset marked by ticks). Decoding scores of stimulus angles (black) and deltas (red) from the simulated population *x* tuned to stimulus angles. The asterisks highlight whether Stim_n_ can be decoded after its offset. The TG matrices correspond to the decoding scores of each decoder trained at time *t* and tested at all time samples. ***C***, More complex networks can be generated by hierarchically connecting feedback loops with one another. Arrows indicate connections within or between the levels of such hierarchy. ***D***, Top, Examples of plausible hierarchies, together with the dynamics of their observable units (*x*) at each hierarchical level (black lines) in response to a brief stimulus and Bottom, The corresponding temporal generalization matrices.

The above results suggest that a series of distinct areas of the visual hierarchy can represent distinct visual stimuli thanks to their cascade organization. To further characterize such macroscopic dynamics, we introduce a modeling framework. Specifically, we formalize the dynamical properties of the underlying cellular mechanisms together with their predictions in terms of decoding and temporal generalization analyses (Fig. 3). The aim of this framework is not to identify the exact cellular mechanisms at play, which is out of reach of EEG analyses, but to estimate the dynamical system underlying these traveling waves.

For example, neuronal adaptation is a cellular mechanism induced by the slow return of sodium and potassium currents that follow a spike. Adaptation temporarily diminishes the expected spiking activity (Fig. 3*A*), and can result in a reactivation of decoding performance after stimulus offset (Fig. 3*B*). Other neuronal mechanisms may lead to macroscopic dynamics indistinguishable from adaption: recurrent connections between excitatory and inhibitory neurons can, for example, lead populations of neurons to decrease their firing after a stimulus onset and to show an rebound effect after its offset. In both cases these adaption and excitatory-inhibitory systems can be simplified as (1) an observable unit *x* [e.g., membrane potential or mean-field activity of postsynaptic potentials (PSP) to excitatory neurons] and (2) a hidden unit *y* (e.g., adaptive ion currents or PSP to inhibitory neurons) connected in a negative feedback loop. More generally, various types of feedback loops (Fig. 3*B*) and feedforward propagations (Fig. 3*C*,*D*) could in principle maintain sensory representations in the EEG activity, even after the offset of the stimulus.

TG analyses can distinguish a variety of such dynamical systems. TG consists in quantifying the extent to which a linear decoder trained at time *t* (across all EEG channels) can accurately decode representations at time *t′* (Stokes et al., 2013; King and Dehaene, 2014). For example, a positive feedback loop input with a brief stimulus leads to a square TG matrix that extends beyond stimulus offset (Fig. 3*C*). By contrast, a negative feedback loop input with the same stimulus leads to a rapid decrease of decodable activity (potentially down to chance level), followed by below-chance decoding after stimulus offset.

Figure 3*D* illustrates that TG can also differentiate more complex dynamical systems. For example, a strictly feedforward architecture leads all decoders trained at a given time sample to generalize for a constant but temporally shifted time period. By contrast, a chain of positive feedback loops (recurrence hierarchy) leads linear decoders to generalize over increasingly longer time periods. Depending on the type of connection between units, a neuronal chain may lead to oscillatory activity (e.g., adaptive hierarchy, Fig. 3*D*) or to positive and negative traveling waves triggered by stimulus onsets and offsets respectively (updating hierarchy, Fig. 3*D*).

### Visual representations propagate across a long chain of negative feedback loops

**Figure 4.**
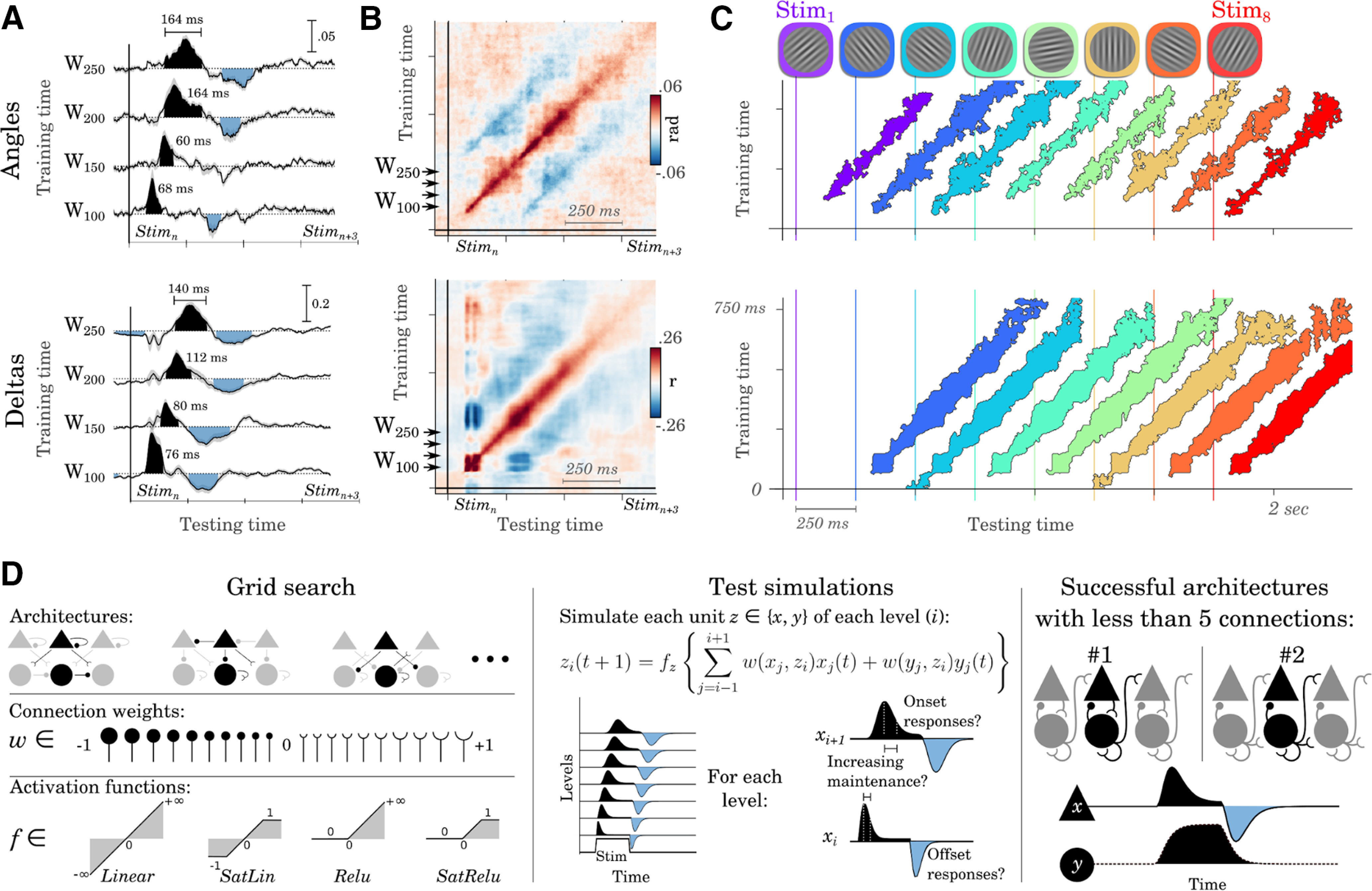
The spatiotemporal dynamics of representations reveal an updating hierarchy. ***A***, Examples of TG for angle (top) and δ (bottom) decoders trained at 100, 150, 200, and 250 ms after stimulus onset and tested across all time samples. The shaded areas indicate significant generalization, cluster corrected across subjects. Time annotations indicate the duration during which each decoder significantly generalized. ***B***, Full TG matrices for angle (top) and δ (bottom) decoders. Blue areas indicate below-chance generalizations. ***C***, TG scores for each of the eight successive stimuli. Colored areas indicate the above-chance generalizations, cluster corrected across subjects. ***D***, Left, Grid-search analyses across architectures, connection weights (*w*) and activation functions (*f*) led to search among >1.5 billion possible hierarchical models. Middle, Each of them was tested on its ability to generate dynamics qualitatively similar to those obtained empirically: that is, characterized by onset and offset responses whose durations increased across levels. Right, Two architectures captured these dynamics with no more than four connections. The plain line illustrates a representative example of an observable unit (*x*). The dotted line illustrates a representative example of the hidden unit (*y*).

Do the hand-picked dynamical systems illustrated in Figure 3 match the spatiotemporal characteristics of the brain responses to visual streams? To address this issue, we separately implemented TG for each subject and verified with nonparametric cluster-level testing across subjects whether each angle and δ decoder trained at a given time sample was able to generalize over all time samples (Fig. 4*A*,*B*). We then tested whether the significant TG clusters matched the predictions of the models illustrated above.

The analyses revealed four main findings. First, both angle and δ TG matrices were characterized by a ∼900 ms-long (above chance) cluster around the TG diagonal (angle, *p* < 0.001; δ, *p* < 0.001). This diagonal pattern invalidates the predictions of nonhierarchical circuits; that is, positive or negative feedback alone do not lead to diagonal TG. Second, the angle and δ decoders successfully generalized, on average, >75 ms (SD = 68 ms) and 129 ms (SD = 54 ms), respectively. These durations are shorter than both (1) the time period during which angles and deltas are decodable (∼900 ms; Fig. 1*D*,*E*, shaded areas) and (2) the stimulus duration (233 ms). These brief generalizations, therefore, invalidate the predictions of the feedforward and positive feedback loop chains as these architectures predict decoders to generalize for a time period longer than the stimulus presentation. Third, these generalization periods consistently increased in duration as a function of training time (angles, *r* = 0.44, *p* < 0.001; deltas, *r* = 0.47, *p* = 0.003; Fig. 4*A*) as predicted by the chains of feedback loops. Fourth, the central positive decoding pattern was surrounded by two below-chance diagonals (*p* < 0.01, for both angles and deltas) time-locked to the stimulus offset (blue areas in Fig. 4*A*,*B*) as predicted by the chain of negative feedback loops. Supplementary analyses confirmed that the reversal of neural responses after stimulus offset only partially accounts for the long-lasting decoding angle and δ scores: specifically, diagonal decoders (i.e., trained and tested at the same time samples) consistently outperformed their reversal counterparts (i.e., trained at time *t* and tested at time *t* + 250 ms, after sign reversal) for both stimulus angle (all *p* < 0.01) and stimulus δ (all *p* < 0.05 but between 750 and 1000 ms).

To test whether these TG patterns were consistently observed for each stimulus, we independently decoded stimulus angles and deltas as a function of their position within the visual sequence. The corresponding TG analyses resulted in a series of parallel and nonoverlapping diagonals (Fig. 4*C*). This result shows that our EEG results can be accounted for by a visual hierarchy multiplexing successive visual representations. Such a cascade can represent multiple events (i.e., a vertical slice, Fig. 4*C*) as well as their respective timing (i.e., a horizontal slice, Fig. 4*C*).

### Systematically searching across dynamical systems confirms this neural architecture

The dynamical systems presented in Figure 3 correspond to a small set of possible neural architectures (Fig. 4*D*). To systematically investigate which architecture could account for subjects' brain activity, we performed a grid search analysis by (1) varying each connection (min = −1, max = 1, step = 0.05) of each architecture as well as by (2) testing four monotonic activation functions (Linear, ReLu, SatLin, SatRelu) for each unit type (*x*, observable; *y*, hidden). Each of the tested networks consisted of a 10-layer hierarchy of *x* and *y* units interconnected with recurrent, feedforward and/or feedback connections (Fig. 4*C*). The resulting models were evaluated on their ability to generate dynamics qualitatively similar to those obtained empirically (see above, Architecture search) while keeping a minimal number of connections (i.e., free parameters). In practice, we implemented the model search efficiently so that simple architectures (i.e., architecture with less connections) are tested before their complex counterparts. After having simulated >1.5 billion models, spanning 531,441 distinct architectures, the search stopped at the level of four connections—that is, the number of parameters that is sufficient and necessary to account for our EEG results. The results showed that only two architectures accounted for subjects' EEG with no more than four connections. The first architecture corresponds to the updating hierarchy (Fig. 4*D*). The second architecture is also a hierarchy of negative feedback loops, within which *x* units are epiphenomenal — i.e., they correspond to leaf nodes and thus do not influence anything else in the network. Importantly, both architectures predict that the hidden units *y* maintain stimulus information over time, whereas the observable units *x* mark the update of these representations.

Overall, these results suggest that, paradoxically, angles and deltas representations are maintained by a biological mechanism undetectable with macroscopic electric brain activity as decoded by a single-sample spatial EEG filter (de Cheveigné and Simon, 2008). The long-lasting decoding scores of these two types of representations does not appear to result from a sustained activity profile but from an observable update signal that propagates across a neuronal chain recruited after the onset and the offset of the stimuli.

## Discussion

The present study makes three main contributions to understanding how the human brain continuously processes its visual influx. First, we confirm that the representations of ∼1 s long typically observed in single-stimulus studies (VanRullen and Macdonald, 2012; Carlson et al., 2013; Cichy et al., 2014; King et al., 2016) can also be observed in the context of visual streams. The discrepancy between the timing of sensation and the timing of neural processing shows that the visual system continuously buffers and updates multiple snapshots of its past stimulations.

Second, both visual content (stimulus angle) and visual flow (stimulus delta) appear to simultaneously propagate across a long chain of neuronal populations, localized along the expected visual hierarchies. This result extends the findings of single-stimulus studies (Lamme and Roelfsema, 2000; Hung et al., 2005; Cichy et al., 2014) to visual streams and suggests that the traveling waves elicited at the onset and offset of the stimulus reflect a series of transient signals. This phenomenon is reminiscent of predictive coding (Rao and Ballard, 1999; Friston, 2018), where bottom-up signals only propagate when sensory information unexpectedly changes. Our model search is consistent with this architecture: the valid models consist of a simple feedforward and locally recurrent hierarchy that propagates information through its observable units *x* when the stimulus changes and otherwise maintains it within its hidden units *y*. Within the limited scope of our model search (i.e., hierarchical modules) and data (i.e., evoked EEG responses), these results are consistent with dynamic coding (Stokes, 2015) activity-silent maintenance theories (Wolff et al., 2017): they suggest that the visual representations of a stimulus are maintained by a mechanism *y* undetectable with single-sample EEG spatial decoders.

Third, our results suggest that low-level visual representations (e.g., stimulus orientation) are redundantly coded in the visual hierarchy. This phenomenon is consistent with recent findings based on the electrophysiological recordings of the inferotemporal cortex of macaques in response to static images (Hong et al., 2016). We can speculate that this redundant coding provides two computational advantages. First, it allows the prefrontal and parietal cortices, which receive inputs from the whole visual hierarchy (Dehaene and Changeux, 2011), to instantaneously bind both (1) multiple levels of representations and (2) multiple successive images and their respective timings with a simple linear readout. Second, this redundant code automatically binds time to each representation: given that different levels of the hierarchy are associated with different synaptic delays and time constants, one can thus linearly read out the relative timing of representation.

The present study faces several limitations. First, it is based on artificial visual streams with a fixed presentation rate and does not explore how natural images presented in movies are processed and maintained. Whether the speed or frequency of stimulus have an impact on cortical traveling waves thus remains outside the scope of the present study. Second, EEG is noisy and thus only provides a lower-bound estimate of the number of successive images simultaneously encoded in brain activity. It is well possible that the brain represents many more successive events at each instant. Third, it is currently unclear whether the relevance of the stimuli can modulate the duration of maintenance. Fourth, the models implemented in the present study are extremely simplistic and only explore hierarchical and modular architecture. The cortex is known to be characterized by recurrent, skip, and top-down connections, whose effects vary with subjects' task and vigilance. Consequently, this study provides a simple and intuitive backbone for further research rather than a full-fledged model of the cortex.

Finally, the present investigation focuses on the neural representations of unexpected stimuli. This approach thus omits the likely critical role of top-down connections. In particular, recent work by Alamia and VanRullen shows that the direction of EEG traveling waves flips from a bottom-up direction to a top-down direction between stimulation and rest, respectively (Alamia and VanRullen, 2019). Although their results also point to a predictive coding architecture, our dynamical modeling remains to be investigated with resting EEG and with expected stimuli.

Overall, the present work contributes to a growing literature on cortical traveling waves (Hughes, 1995; Nunez and Cutillo, 1995; Ermentrout and Kleinfeld, 2001; Nunez and Srinivasan, 2006; Klimesch et al., 2007). In particular, previous studies have shown that cortical gamma activity was coordinated by traveling in the alpha frequency range (Bahramisharif et al., 2013) and can be evoked by eye movements (Giannini et al., 2018). In addition, traveling waves have been shown previously to carry specific representations. For example, Benucci et al. (2007) showed with cat electrophysiology that within *V–1*, the orientation of a stimulus elicited specific standing waves, whereas the spatial position of a stimulus elicited local traveling waves propagating at 0.2–0.5 m/s^–1^ within *V1*. In addition, Sauseng et al. (2002) have shown that evoked theta oscillations spread from front to back during retrieval attempt and then reverses direction once the information is retrieved. Here, we complement these findings by showing that multiple macroscopic waves, each carrying different visual representations, simultaneously travel across the visual hierarchy to continuously transform the avalanche of sensory input into a coherent stream of mental representations.
